# Laboratory management for SARS-CoV-2 detection: a user-friendly combination of the heat treatment approach and rt-Real-time PCR testing

**DOI:** 10.1080/22221751.2020.1775500

**Published:** 2020-06-18

**Authors:** Fabiola Mancini, Fabrizio Barbanti, Maria Scaturro, Giulia Errico, Angelo Iacobino, Antonino Bella, Flavia Riccardo, Giulia Marsili, Paola Stefanelli, Patrizio Pezzotti, Giovanni Rezza, Alessandra Ciervo, on behalf ISS COVID-19 study group

**Affiliations:** a Department of Infectious Diseases, Istituto Superiore di Sanità, Rome, Italy; b European Public Health Microbiology Training Program (EUPHEM), European Centre for Disease Prevention and Control (ECDC), Stockholm, Sweden

**Keywords:** SARS-CoV-2, COVID 19, rt-Real time PCR, molecular methods, heat treatment, nasopharyngeal samples

## Abstract

The RNA purification is the gold standard for the detection of SARS-CoV-2 in swab samples, but it is dependent on the availability of chemical reagents. In this study, we evaluated the heat treatment method without RNA extraction as a reliable option to nucleic acid purification.

ISS COVID-19 study group: Angela Di Martino, Marzia Facchini, Simona Puzelli, Giuseppina Di Mario, Laura Calzoletti, Stefano Fontana, Stefano Fiore, Antonella Marchi, Eleonora Benedetti, Concetta Fabiani, Giulietta Venturi, Claudia Fortuna, Antonello Amendola, Laura Villa, Daniela Fortini, Marco Di Luca, Luciano Toma, Francesco Severini.

From December 2019, several pneumonia cases of unknown etiology were reported in Wuhan city, Hubei Province, China, and were epidemiologically linked to the Huanan Seafood Wholesale Market where live animals were also sold [1]. In January 2020, the infectious agent was identified by gene sequencing and named provisionally 2019-nCoV [2]. Subsequently, the International Committee on Taxonomy renamed as SARS-CoV-2 the virus responsible for the coronavirus disease 19 (COVID-19), because it was found closely related to severe acute respiratory syndrome coronavirus (SARS-CoV) [3,4]. The SARS-CoV-2 sequence shared in the GISAID platform, allowed the development of several molecular diagnostic tests through specific primers and probes design for the rapid detection by reverse transcription (rt) Real-time PCR [5,6]. In the course of infection, SARS-CoV-2 is usually detectable in upper and lower respiratory specimens and nasopharyngeal secretions collected using swabs are the clinical samples of choice for diagnostic testing [7,8]. Emergency response to the COVID-19 pandemic has highlighted the critical need of making reliable diagnostic ascertainment widely available, in order to favour the rapid detection and isolation of cases and investigation, monitoring and quarantine of their close contacts. These measures in turn are aimed at reducing the risk of onward transmission within communities [9].

In this perspective, to overcome a possible health crisis, different commercial diagnostic kits were promptly developed and introduced into the market before their analytical and clinical validation (https://www.finddx.org/covid-19/pipeline). As these kits are routinely used in clinical laboratories, there is a risk of generating false-negative or false-positive results. On the other side, demand for commercial RNA extraction kits, especially for robotic platforms, has increased enormously worldwide creating a serious problem of shortage. Whit the pandemic spread of SARS-CoV-2, in the absence of effective drugs and vaccines, the “test, track, trace” strategy will be foremost global public health response option for several months. As this approach pivots on broad testing capacity, it is critical to develop alternative diagnostic workflows, as current ones that are dependent on diagnostic reagents that are prone to stock outs. In this study, we evaluated the performance of the heat treatment approach against currently recognized gold standards for RNA purification for laboratory confirmation of COVID-19 cases in order to assess its sensitivity and specificity. By testing a method that does not require RNA extraction, the overall aim of our work was to provide a feasible alternative to current method that requires chemical reagents that are in increased demand as many countries approach the phase 2 of the COVID-19 pandemic.

Positive SARS-CoV-2 nasopharyngeal swabs (Copan Diagnostics, Brescia, Italy) in Universal Transport Medium (UTM) were used to set up the method. Undiluted and diluted samples in H_2_O (1:5, 1:10, 1:20) were thermally treated in dry bath at different combination of temperature (70°C, 80°C, 90°C, 95°C and 98°C) and time of incubation (3, 5, 10 and 15 min). Samples were then placed in ice for 5 min, centrifuged at 1000*g* for 1 min and the supernatant was used for molecular assays. Comparably, the same undiluted and diluted samples were subjected to RNA extraction using QIAamp viral RNA mini kit (Qiagen, Hilden, Germany), eluted in H_2_O in the original sample volume and kept in ice. Samples (5 µl/reaction) were analysed for N1, N2 and RP genes by in-house rt-Real-time PCR through the LC480 II instrument (Roche) using reagents and protocol from CDC (Division of Viral Diseases, Centers for Disease Control and Prevention-USA) [10]. Subsequently, 30 negative and 60 positive samples, randomly selected and distributed in different Cycle threshold (CT) ranges, were molecular tested for the presence of SARS-CoV-2 using the heat protocol vs. purified RNA extraction by in-house rt-Real-time PCR and with the commercial 2019-nCoV TaqMan RT-PCR Kit (Norgen Biotek Corp., Canada), according to the manufacturer’s protocol. Thermal treated and purified RNA samples were also stored at −20°C. All rt-Real-time PCR reactions were run in triplicate and in two independent runs, for intra- and inter-assay reproducibility, respectively. Finally, to evaluate the performance of molecular assays a standard curve was generated by 10-fold dilutions of SARS-CoV-2 RNA, isolated and extracted at Istituto Superiore di Sanità in Rome, Italy, and quantified by a well-established copy number of RNA synthetic E gene (Wuhan coronavirus, EVAg, www.european-virus-archive.com) [11].

The evaluation of several temperature and time of incubation provided evidence that the best combination was the treatment at 95°C for 10 min with a dilution of 1:10 of sample. Molecular analyses showed no statistically significant difference regard the ΔCT value among N1, N2 and RP target genes (*P* > 0.5). In contrast, rt-Real-time PCR indicated a ΔCT of 9.62 (range 10.5−9.0 ± SD 5.22) for undiluted samples, and a ΔCT of 5.3 (range 6.6−4.3 ± SD 3.01), 0.6 (range 1.1−0.0 ± SD 0.42) and 0.4 (range 0.9−0.3 ± SD 0.41) for 1:5, 1:10 and 1:20 H_2_O diluted swabs, respectively. These findings demonstrated that the direct and 1:5 diluted samples are not suitable for PCR analyses, probably due to the presence of inhibitors in the UTM medium such as gelatin or sucrose. No significant difference was observed between 1:10 and 1:20 diluted samples, guiding the choice for the 1:10 dilution.

The heat treatment protocol was applied to 30 negative and 60 positive samples with recorded CT values. All specimens were also manually extracted and tested for the presence of SARS-CoV-2 by in-house rt-Real-time PCR and the 2019-nCoV TaqMan RT-PCR Kit. In particular, we investigated the RNA availability and virus detection using both the purified and thermal/non-extractive procedures also with this commercial kit because it is based on the same primers, probes and assays developed by the CDC and used in the in-house molecular method.

Both molecular approaches were assessed by a standard curve generated by SARS-CoV-2 RNA, using 10-fold serial dilutions of the viral RNA ranging from 1 ×10^6^–10 copies/µl. An inverse linear relationship (*y* = −3.427× + 41.87; *R*^2^ = 0.0309) was generated by plotting crossing points values against artificial E gene concentration. The linear range spanned CT values ranging from 20.5 ± SD 0.1 to 36.9 ± SD 0.5, corresponding to concentrations of 10^6^ to 10 copies per μl, respectively. No difference was found among gene targets and results from in-house and commercial rt-Real Time PCR were reported for N2 gene (Table 1). Compared with purified RNA, the in-house protocol showed a 100% of sensitivity and specificity for heat-treated samples, whereas the commercial Kit exhibited a 100% of sensitivity and specificity for extracted method and 86.67% of sensitivity and 100% specificity for the heat protocol (Table 1). The positive detection of the in-house system ranged from 4.5 × 10^6^ to 15 copies/reaction and from 3.1 × 10^6^ to 40 copies/reaction for extracted RNA and in inactivated samples, respectively. A lower efficiency was observed with the commercial kit where it was registered a range of 1.2 × 10^6^ to 70 copies/reaction for purified samples and 4.5 × 10^5^ to 80 copies/reaction for heat samples. It should be noted that the preliminary 10^−1^ dilution factor might influence the tendency for increased CT values and the copy number detection. This observation is stressed especially in heat-treated samples with CT ≥ 35 and may generate false-negative results in low viral load specimens (Table 1). All purified and heat-treated samples maintained at −20° C were tested again after 96 h with both rt-Real Time protocols to investigate a possibly less sensitivity with regard to samples directly processed after storage. No significant variation was observed (data not shown), suggesting a feasible preservation of specimens before the molecular assay.

**Table 1.** Distribution of CT value results generated by N2 gene target from extracted and heat-treated samples.

Extracted samples^ab^	Heat-treated samples^b^ *n* (mean CT ± SD)^d^		Extracted samples^c^ *n* (mean CT ± SD)^d^		Heat-treated samples^c^ *n* (mean CT ± SD)^d^	
Positive (*n* = 60)						
CT range (*n*/mean CT ± SD)^d^	Positive	Negative	Positive	Negative	Positive	Negative
15–20 (5/19.6 ± 0.3)	5 (19.6 ± 0.3)	0	5 (19.5 ± 0.3)	0	5 (19.9 ± 0.2)	0
20–25 (12/23.2 ± 1.2)	12 (24.2 ± 1.2)	0	12 (22.9 ± 1.3)	0	12 (24.5 ± 1.1)	0
25–30 (18/27.6 ± 1.4)	18 (28.6 ± 1.3)	0	18 (27.0 ± 1.9)	0	18 (32.1 ± 1.0)	0
30–35 (15/33.2 ± 1.3)	15 (33.8 ± 1.1)	0	15 (32.4 ± 1.3)	0	15 (34.4 ± 1.0)	0
>35 (10/36.3 ± 0.8)	10 (37.2 ± 1.1)	0	10 (35.7 ± 0.6)	0	2 (35.7 ± 0.2)	0
Negative (*n *= 30)	0	30	0	30	8	30
	Sensitivity (%) [95% CI] 100% [100%–100%] Specificity (%) [95% CI] 100% [100%–100%] PPV^e^ (%) [95% CI] 100% [100%–100%] NPV^e^ (%) [95% CI] 100% [100%–100%]		Sensitivity (%) [95% CI] 100% [100%–100%] Specificity (%) [95% CI] 100% [100%–100%] PPV^e^ (%) [95% CI] 100% [100%–100%] NPV^e^ (%) [95% CI] 100% [100%–100%]		Sensitivity (%) [95% CI] 86.67% [79.64%–93.69%] Specificity (%) [95% CI] 100% [100%–100%] PPV^e^ (%) [95% CI] 100% [100%–100%] NPV^e^ (%) [95% CI] 78.95% [70.52%–87.37%]	

^a^Method chosen as reference.

^b^In-house rt-Real Time PCR.

^c^2019-nCoV TaqMan RT-PCR Kit (Norgen Biotek Corp., Canada).

^d^Only positive results are included in the calculation of mean CT.

^e^(PPV) Positive predictive value; (NPV) Negative predictive value.

The proposed heat approach protocol could also produce similar results using the E gene test described by Corman et al. Moreover, comparing the performance of SARS-CoV-2 protocols described by Corman et al. and developed from CDC, N2 and E primer/probe sets were found to be more sensitive than others [12,13]. A recent study described a similar heat-processed protocol (98°C for 5 min) directly from swab saline/transport solution without any dilution. This study corroborates our results for in-house rt-Real Time PCR, showing a lower sensitivity of the heat treatment (range ΔCT value of 0.5-1.0) when compared with purified samples, but, dissimilar to our findings, a total inhibition was found by the commercial kit RealStar SARS-CoV-2 RT-PCR (Altona Diagnostics, Hamburg, Germany), where all positive samples failed in the detection of Sars-CoV-2 [14]. Probably, in our study, the 1:10 dilution before the thermal approach might decrease the concentration of inhibitors allowing the PCR amplification of specific target.

Compared to the extraction method, the heat treatment assay allows the testing of clinical samples within a very short time (3–3.30 h vs. 5.30–6 h), but more importantly it does not require RNA extraction and is viable if chemical extraction kits are not available. Moreover, it is possible with this method to store samples at −20°C without affecting the performance of the molecular test, if testing needs to be deferred.

The main limit of this method is that the lack of concentration reduces its sensitivity and might not detect subject with very low viral loads. Therefore, it is best applied to testing samples from patients with active infection during which high viral loads are expected. In this context, it is an easy, rapid and most of all universally available alternative procedure. This molecular test should be performed by trained laboratory personnel who are proficient in carrying out Real-Time PCR assays. With respect to negative results, in general it should be considered that those samples must be processed by a viral extraction method. Future experiments will be need to examine whether the heat protocol can be applied to other specimen types and should be compared with other commercial kit tests.
